# Contemporary approach to cardiogenic shock care: a state-of-the-art review

**DOI:** 10.3389/fcvm.2024.1354158

**Published:** 2024-03-13

**Authors:** Aditya Mehta, Ilan Vavilin, Andrew H. Nguyen, Wayne B. Batchelor, Vanessa Blumer, Lindsey Cilia, Aditya Dewanjee, Mehul Desai, Shashank S. Desai, Michael C. Flanagan, Iyad N. Isseh, Jamie L. W. Kennedy, Katherine M. Klein, Hala Moukhachen, Mitchell A. Psotka, Anika Raja, Carolyn M. Rosner, Palak Shah, Daniel G. Tang, Alexander G. Truesdell, Behnam N. Tehrani, Shashank S. Sinha

**Affiliations:** ^1^ Department of Cardiovascular Disease, Inova Schar Heart and Vascular, Inova Fairfax Medical Campus, Falls Church, VA, United States; ^2^ Department of Cardiovascular Disease, Virginia Heart, Falls Church, VA, United States; ^3^ Department of Medicine, University of Texas Southwestern Medical Center, Dallas, TX, United States

**Keywords:** acute myocardial infarction, heart failure, cardiogenic shock, pulmonary artery catheter, shock team

## Abstract

Cardiogenic shock (CS) is a time-sensitive and hemodynamically complex syndrome with a broad spectrum of etiologies and clinical presentations. Despite contemporary therapies, CS continues to maintain high morbidity and mortality ranging from 35 to 50%. More recently, burgeoning observational research in this field aimed at enhancing the early recognition and characterization of the shock state through standardized team-based protocols, comprehensive hemodynamic profiling, and tailored and selective utilization of temporary mechanical circulatory support devices has been associated with improved outcomes. In this narrative review, we discuss the pathophysiology of CS, novel phenotypes, evolving definitions and staging systems, currently available pharmacologic and device-based therapies, standardized, team-based management protocols, and regionalized systems-of-care aimed at improving shock outcomes. We also explore opportunities for fertile investigation through randomized and non-randomized studies to address the prevailing knowledge gaps that will be critical to improving long-term outcomes.

## Introduction

Cardiogenic Shock (CS) is a multifactorial, hemodynamically complex syndrome characterized by profound and refractory circulatory collapse due to impaired myocardial contractility resulting in systemic hypoperfusion, metabolic acidosis, and refractory multiorgan system dysfunction (1). Despite more than two decades of advances in interventional techniques, the development of rapidly deployable temporary mechanical circulatory support (tMCS) devices, and systems-of-care strategies to treat AMI, patients with CS continue to fare poorly with 30%–50% risk of 30-day mortality and associated multiorgan dysfunction in the modern era (2–8). It was not until the landmark Should We Emergently Revascularize Occluded Arteries in Cardiogenic Shock (SHOCK) trial in 1999, however, that a strategy of early revascularization was demonstrated to improve outcomes (9, 10). While randomized controlled trials (RCTs) have failed to demonstrate clear benefit with either pharmacologic or device-based therapies, burgeoning observational research from North American CS registries have highlighted the potential merits of standardized, team-based care in improving the care of patients with CS (11–14). These studies suggest that the implementation of a multidisciplinary “Shock team,” employing early utilization of pulmonary artery catheter (PAC) hemodynamic monitoring, selective and hemodynamically tailored tMCS and comprehensive longitudinal care in an American Heart Association (AHA) Level 1 cardiac intensive care unit (CICU) may be associated with reduced in-hospital mortality. The heterogeneity of clinical presentations, complex hemodynamic perturbations, therapeutic strategies, and eventual outcomes delineates the inadequacy of a one-size-fits-all CS management algorithm. In this narrative review, we discuss the pathophysiology of CS, the phenotypes and evolving definitions to risk stratify this disease state, the gamut of pharmacologic and device-based therapies currently available, and potential treatment protocols and systems-of-care strategies to enhance outcomes in this condition. Finally, we discuss opportunities for fertile investigation to address the prevailing knowledge gaps that persist in this disease.

### Epidemiology of cardiogenic shock

CS remains the most common type of shock in patients admitted to the CICU in the contemporary era (15, 16). Historically, CS was seen as a primary disorder caused by LV dysfunction resulting from AMI. However, in recent years, acute decompensation of chronic HF has been recognized as the most common underlying etiology, contributing up to >50% of the admissions for CS (15). Numerous single and multi-center studies have reported the increasing prevalence of non-AMI CS (15–19). This paradigm shift may be attributed to increased use of preventive therapies and the concomitant decline in AMI incidence, early revascularization strategies in AMI patients leading to decreased incidence of resultant CS, and improved AMI survival in patients sustaining significant irreversible ischemia, resulting in more survivors with chronic HF due to LV dysfunction (20, 21). Patients are now living longer because of declining mortality from AMI-CS (18, 19). Among 1,254,358 CS admissions from the National Inpatient Sample (NIS) from 2004 to 2018, there was a more than threefold increase in the number of hospitalizations accompanied by a substantial 25% reduction in in-hospital mortality (from 49% to 37%) during the study period (22).

Healthcare costs have burgeoned revealing the inexorable financial toll of CS on both individual patients and healthcare systems. In one retrospective observational study of the NIS population from 2000 to 2014, Vallabhajosyula et al. reported a median cost of hospitalization of $80,346 for AMI-CS and $183,767 for CS-associated multiorgan failure hospitalizations (23). A single-center retrospective study of 230 patients reported the cost of AMI-CS hospitalization was more than five times that of AMI without CS, mostly (80%) due to the use of invasive procedures and device usage in the ICU (24). The financial outlay extends beyond the index hospitalization phase, as higher CS—associated readmission rates and progressive disease result in costly follow-up care. Re-hospitalizations contribute to the already elevated cost burden, with 30-day readmission rates nearing 19% and a median cost of nearly $10,000 per readmission (25).

### Pathophysiology of cardiogenic shock

The pathophysiology of CS is a complex and vicious cycle often culminating in multiorgan failure and death. Initiated by a progressive impairment in ventricular contractility, CS leads to a critical reduction in mean arterial pressure (MAP) and cardiac output (CO), resulting in systemic hypoperfusion and decreased coronary perfusion pressure with compensatory activation of baroreceptors and chemoreceptors in an attempt to maintain hemodynamics and perfusion (1, 26, 27). Arterial- and veno-constriction occur as a result of baroreceptor and chemoreceptor activation, causing an increase in vascular resistance with blood distribution away from splanchnic circulation and elevating pulmonary venous and central venous pressure (CVP); these mechanisms result in multiorgan congestion, often exacerbating preexisting volume overload seen in patients with HF, and further compromise end-organ perfusion represented by worsening lactic acidemia (28, 29). In response to tissue ischemia, a state of systemic inflammatory response syndrome (SIRS) ensues which leads to systemic vasodilation and inflammation in an already dysfunctional myocardium—propagating the progressive maladaptive spiral of CS (11, 30).

The two most commonly recognized etiologies of CS are AMI-CS and HF-CS (11). AMI-CS is typically associated with injury to >40% of the LV myocardium but can also be precipitated by mechanical complications such as ventricular septal defect and free wall or papillary muscle rupture (31). Analysis of pressure-volume loop curves in AMI-CS show a rightward and downward shift of the end-systolic pressure volume relationship suggesting a sudden reduction in LV contractility resulting in reduced stroke volume (SV), CO, and MAP and increases in pulmonary capillary wedge pressure (PCWP) and CVP (26, 32). These hemodynamic changes reflect the canonical clinical course for patients with AMI-CS, often beginning with hypotension from the acute ischemic insult leading to hypoperfusion and culminating with congestion. In contrast, HF-CS often follows a more indolent clinical course, usually presenting with congestion in acute on chronic HF-CS phenotypes leading to hypoperfusion and ending with systemic hypotension (11, 26).

### Definitions and classifications of cardiogenic shock

Despite varying definitions in clinical trials, CS has been historically described as a state of systemic hypoperfusion due to impaired myocardial contractility, typically with associated hypotension (systolic blood pressure <90 mmHg for a prolonged duration usually extending >30 min) (2, 4, 9). Patients may also clinically present in a state of isolated hypoperfusion, in which they are normotensive due to compensatory system vasoconstriction, but may still have hemodynamic and biochemical evidence of malperfusion (33). This underrepresented and poorly studied patient population was first reported in the SHOCK Trial registry with a 43% in-hospital mortality (34). Recognizing the need to evolve from binary determination of CS, which does not adequately describe the spectrum and myriad of etiologies and phenotypes or the varied clinical presentations of shock, a multidisciplinary workgroup at the Society for Cardiovascular Angiography and Intervention (SCAI) established a five stage (A to E) classification system for CS in 2019, encompassing the full spectrum of the syndrome based on physical examination findings, laboratory markers, and invasive hemodynamics (35). The SCAI classification system has undergone retrospective and prospective validation in several single center and multicenter registries, with the most recent iteration published in 2022 emphasizing the presence of cardiac arrest with coma as an adverse effect modifier, dynamic baseline and maximum SCAI staging and serial re-staging, and treatment intensity to stratify risk (7, 36–38).

In the SCAI taxonomy, each stage of CS severity is defined by biochemical, physical exam, and hemodynamic findings with the development of tissue hypoperfusion, end-organ dysfunction, and the need for hemodynamic support heralding the transition from pre-shock (Stage B) to later stages (C-E) (35). In 2022, SCAI proposed the addition of a 3-axis model of CS evaluation and prognostication to SCAI staging, recognizing the dynamic nature of shock, and serving as a reminder to individualize patient care based on risk modifiers, clinical phenotype, and comorbidities (37).

More recently, the Cardiogenic Shock Working Group (CSWG) has proposed a pragmatic revision to the SCAI classification to allow for patient risk-stratification using consistent and uniform definitions (36). Utilizing data from 3,455 patients across 17 hospitals from 2016 to 2021, the SCAI-CSWG classification provides specific threshold values to define hypotension (SBP and MAP) and hypoperfusion [lactate, alanine transaminase (ALT), pH] across all stages, and incorporates other relevant variables including treatment intensity (number of vasoactive agents, inotropic therapy, and acute MCS devices) and out-of-hospital cardiac arrest (OHCA). In brief, under the newly refined classification, SCAI-CSWG Stage A is a broad representation of a myriad of hemodynamically stable patients with known cardiac diagnoses which place them at risk for CS. SCAI-CSWG Stage B patients are defined as having either isolated hypotension (SBP 60–90 mmHg or MAP 50–65 mmHg) or hypoperfusion (lactate 2–5 mmol/L or ALT 200–500 U/L) without the need for drug or device therapy. SCAI-CSWG Stage C represents the more “classic CS” patients with hypoperfusion (lactate 2–5 mmol/L or ALT 200–500 U/L) and hypotension (SBP 60–90 mmHg or MAP 50–65 mmHg) or requiring one drug or tMCS device. SCAI-CSWG Stage D patients represent those deteriorating despite initial therapies who remain hypotensive (SBP 60–90 mmHg or MAP 50–65 mmHg) with signs of worsening hypoperfusion (lactate 5–10 mmol/L or ALT >500 U/L) requiring 2–5 treatment-intensive drugs or tMCS devices or have persistent hemodynamic instability on one drug or device. Lastly, SCAI-CSWG Stage E, or extremis shock, represent patients with (1) refractory hypotension (SBP <60 mmHg or MAP <50 mmHg), (2) refractory hypoperfusion (lactate >10 mmol/L), (3) requiring >3 drugs or tMCS devices, or (4) suffering an OHCA with coma. One significant difference between the original proposed SCAI system and the new SCAI-CSWG nomenclature centers around the definition of Stage B (beginning) shock, the latter allowing for earlier recognition and more reliable capture of patients with normotensive hypoperfusion—a largely unrecognized entity increasingly identified as an independent risk factor for CS mortality (36). In this manner, the SCAI-CSWG classification provides a granular, uniform taxonomy for risk stratification to delineate the temporal progression of CS across SCAI Stages (36).

Invasive hemodynamic assessment may also be useful in the classification of CS. The original Diamond-Forrester nomenclature initially proposed binary classification of HF patients with respect to perfusion and congestion based on the initial cardiac index and pulmonary capillary wedge pressure (PCWP) assessments, respectively (39–41). Modern day hemodynamic profiling of CS has become more nuanced, further classifying CS with distinct congestive profiles including “LV-dominant”, “RV-dominant”, and “biventricular (BiV)” shock profiles (33). LV-dominant CS is characterized by reduced LV function with elevated PCWP and normal or reduced CVP, while RV-dominant CS is characterized by elevated CVP with relatively preserved LV function and normal to low PCWP and pulmonary artery pressure. Lastly, as the name suggests, BiV shock is characterized by reduced LV function with elevated right and left cardiac filling pressures (1, 35). A sub-study of SHOCK registry reported similar in-hospital mortality in patients with RV vs. LV shock (53% vs. 61%, *p* = 0.30) (42). However, recent literature suggests that BiV shock is not only the most commonly observed profile out of the three congestive entities but also is a significant independent predictor of mortality when compared to LV congestion profile [aOR 2.4 (95% CI 1.4–3.7)] or no congestion [aOR 2.1 (95% CI 1.1–4.0)], and not necessarily driven by RV predominant shock (43).

### Acute myocardial infarction related cardiogenic shock (AMI-CS)

With an aging population and associated frailty syndromes, up to 10% of patients presenting with AMI are likely to not only have profound hemodynamic perturbations, but also complex coronary artery disease (CAD), to include multivessel disease, chronic total occlusions and calcified vasculature (2, 44, 45). In patients with pre-existing CAD, even small ischemic injuries can precipitate CS, jeopardizing up to 40% of myocardial mass (46). Initially coined a “downward spiral” by Hollenberg in 1999, the central pathophysiologic premise of AMI-CS is an ischemic insult from an acute coronary thrombosis resulting in regional myocardial necrosis, impaired cardiac output due to concomitant systolic and diastolic dysfunction, elevated intracardiac filling pressures and reflexive sympathetic activation resulting in systemic vasoconstriction and heightened afterload (47). The timing of CS onset following AMI is variable, with a post-hoc analysis of the SHOCK Trial registry reporting a median time from AMI symptoms to CS onset of 6 h, with the greatest delay in patients in which the left anterior descending artery was the culprit vessel (48). Up to 20% of patients with AMI-CS may also develop refractory and non-infectious SIRS, due to the upregulation of nitric oxide synthetase and associated cytokines (49). These patients are at increased risk for non-cardiac organ dysfunction as well as higher in-hospital and one-year mortality rates across the CS severity spectrum. Lastly, patients with AMI may develop CS due to mechanical complications, such as ventricular septal defects, papillary muscle rupture with consequent severe mitral regurgitation, and free wall rupture (50). The landmark SHOCK Trial fundamentally altered our understanding and treatment approach to AMI-CS after it demonstrated a 13% absolute reduction in all-cause mortality at one year in patients undergoing revascularization (10). The prognostic relevance of timely invasive reperfusion in this patient population was further reinforced by the findings of the Feedback Intervention and Treatment Times in ST-Elevation Myocardial Infarction (FITT-STEMI) trial which demonstrated every 10 min treatment delay was associated with 3 additional deaths per 100 patients with AMI-CS undergoing percutaneous coronary intervention (PCI) (51). The classic term “golden hour” was coined by R. Adams Cowley in 1975 where he stated, “the first hour after injury will largely determine a critically injured person’s chances for survival.” This “golden hour” in CS management must include prompt identification followed by timely revascularization, resuscitation, and admission to CICU for escalating levels of care as appropriate (52). While the term “door to balloon” (D2B) is pertinent for ST-elevation myocardial infarction (STEMI) cases, in the realm of CS, the analogous “shock to support” (S2S) and “shock to perfusion” (S2P) durations have yet to be established. These timelines reflect the window for restoring adequate blood pressure and preventing multiorgan failure, comparable to the urgency of re-establishing coronary blood flow in STEMI patients. Basir et al. demonstrated the impact of early intervention on associated survival rates. Initiating tMCS within 1.25 h of shock onset yielded a survival rate of 66% in contrast to 26% when tMCS was initiated beyond 4.25 h (53). Despite advances in interventional therapies and systems-of-care strategies for the treatment of STEMI, AMI-CS continues to pose a challenge to health systems and clinicians worldwide given its multiorgan system ramifications (54).

### Emergency department care

Prompt recognition of CS by emergency medical services (EMS) personnel and emergency department providers is key to ensuring timely treatment of patients with AMI with circulatory collapse. Steps to expedite the care of AMI-CS include early 12-lead electrocardiogram acquisition, administration of vasopressors (preferably norepinephrine and avoidance of phenylephrine) to achieve MAP >65 mm Hg, mechanical ventilation, point-of-care echocardiography to assess for mechanical complications, and immediate transfer to a primary PCI-capable facility (55–58). While patients with SCAI Stages A or B AMI-CS may proceed directly to cardiac catheterization laboratories (CCL), those with SCAI stage C or D may first require adjunctive stabilization measures while also mitigating any significant delays to invasive reperfusion (59). For patients with SCAI stage E AMI-CS, particularly those without ST-segment elevation, and who may have had prolonged out-of-hospital cardiac arrest, refractory ventricular arrhythmias and severe lactic acidosis with unfavorable prognosis, careful multidisciplinary team review of expected prognosis and elucidation of prior patient wishes and goals of care is warranted, often followed by formal palliative care consultation (60, 61).

### Best practices for vascular access

Transradial access has now been recognized as the default approach for coronary angiography and PCI for patients with acute and chronic coronary syndromes following clinical trial data demonstrating reductions in major bleeding and vascular complications as compared to the femoral approach (62–64). These findings have been similarly noted in patients with AMI-CS (65). It is recognized, however, that AMI-CS is an important predictor of transradial access failure, as these patients have systemic constriction and are often receiving vasoactive therapies. They may also concomitantly require large bore access to facilitate implantation of tMCS (66). Therefore, if radial access is challenging or tMCS is required, concerted efforts should be made to employ safe femoral access using contemporary multimodality imaging techniques (67). The core elements of “vascular safety bundles” include routine ultrasound and fluoroscopic guided micropuncture access, pre-and post-procedure run-off angiography, and validated hemostatic protocols (68). In select patients with peripheral vascular disease or severely constricted lower extremity vessels, further measures may be undertaken to mitigate the risk for bleeding and vascular complications. These include upstream SHiP (Single access for High Risk PCI) in patients supported with Impella CP (Abiomed, Danvers, MA) and use of distal perfusion catheters in patients requiring downstream tMCS who are at risk for acute limb ischemia (68, 69).

### Antithrombotic therapy

Multiple factors pose a challenge to achieving prompt, safe, and consistent antithrombotic effects in AMI-CS including but not limited to: (1) impaired absorption of oral P2Y12 inhibitors due to microcirculatory dysfunction and opioid induced enteral dysmotility; (2) platelet dysfunction due to hypothermia during targeted temperature control (TTC), microcirculatory thrombosis, myeloid dysfunction and acute renal failure; (3) impaired cytochrome P450-dependent activation of clopidogrel due to hepatosplanchnic malperfusion; and (4) bleeding and vascular complications due to large bore access (59). To date, recommendations for antiplatelet and antithrombotic treatments in AMI-CS have been extrapolated from patients with hemodynamically stable acute coronary syndrome, given limited efficacy data in CS (70). Given the high burden of hepatic and renal failure in CS with associated risk for inconsistent pharmacokinetics and pharmacodynamics, the AHA Position Statement on CS and European guidelines recommend the use of unfractionated heparin as the anticoagulant of choice due to its rapid offset and reversibility (1, 71).

Dual antiplatelet therapy (DAPT) with aspirin and oral P_2_Y_12_ inhibition is the mainstay of contemporary antiplatelet therapy in patients with AMI. The newer generation P_2_Y_12_ inhibitors prasugrel and ticagrelor, in crushed formulations, are generally the preferred oral agents because of their rapid onset of action and favorable pharmacodynamics compared to clopidogrel (72–75). In circumstances where oral bioavailability may be limited, use of the intravenous P_2_Y_12_ inhibitor cangrelor to bridge the gap in platelet inhibition may be warranted. With the ability to achieve steady state concentration within 2 min of infusion and a half-life of less than 6 min, cangrelor has been studied in cardiac arrest patients and has been demonstrated to impart more consistent platelet inhibition without increased risk for bleeding compared to oral agents (76). The Dual Antiplatelet Therapy for Shock Patients with Acute Myocardial Infarction trial (DAPT-SHOCK-AMI) (ClinicalTrial.gov: NCR03551964) is an ongoing multicenter randomized study comparing the primary clinical and laboratory endpoints of 30-day death/myocardial infarction/stroke and ADP-induced platelet aggregation between cangrelor and ticagrelor in AMI-CS. There is limited data regarding the clinical utility of glycoprotein IIb/IIIa inhibitors in AMI-CS, with concurrent class IIa recommendation in select non-CS AMI patients with high thrombus burden, no-reflow phenomenon or abrupt periprocedural vessel closure (63, 77).

### Revascularization strategy

Up to 80% of patients with AMI-CS will have multivessel CAD, an independent predictor of morbidity and mortality in this patient population (78). Despite clinical precedent favoring coronary artery bypass grafting (CABG) in patients with ischemic left ventricular dysfunction, less than 7% of shock patients undergo surgical revascularization given their elevated risk for perioperative morbidity and mortality (21, 79). Notwithstanding the merits of complete revascularization in patients with hemodynamically stable acute coronary syndromes, gaps remain in knowledge regarding the extent of revascularization strategies that most benefit patients with AMI-CS (80). To date, only one large, multicenter randomized clinical trial has addressed this issue. The Culprit Lesion Only PCI vs. Multi-vessel PCI in Cardiogenic Shock (CULPRIT-SHOCK) trial demonstrated a 17% absolute reduction in the primary endpoint of 30-day death or renal replacement therapy with culprit-vessel PCI (2). Similar findings were noted at one year, with the caveat that the culprit revascularization cohort had higher rates of heart failure rehospitalization and repeat revascularization during this intermediate time period (81). The applicability of this study’s findings to real world practice has been challenged, as fewer than one-third of all patients had tMCS and nearly one-quarter of the multivessel cohort also underwent PCI of coronary chronic total occlusions, a practice that has not been demonstrated to improve cardiac function in non-CS patient with acute coronary syndrome (82). A recent sub-study of the National Cardiogenic Shock Initiative (NCSI), for instance, showed no differences in in-hospital mortality, acute kidney injury and length of stay in patients with AMI-CS undergoing multivessel PCI when Impella was implanted prior to revascularization (83). The US and European guidelines have nevertheless downgraded the practice of ad-hoc multivessel PCI in AMI-CS to a Class III recommendation based on the CULPRIT-SHOCK findings (63, 64). Similarly, there is a paucity of data regarding coronary stent platforms in AMI-CS (84). However, iterative advances in drug elution pharmacokinetics and emerging data suggest ischemic equipoise with attenuated bleeding risk in select high-risk patients undergoing PCI; drug-eluting stents are preferred over bare-metal stents in patients undergoing PCI (85).

### Heart failure related cardiogenic shock (HF-CS)

In the last decade, HF-CS has been recognized as a distinct etiology which varies from AMI-CS not only in terms of pathophysiology and clinical presentation but also with respect to acute management and long-term prognosis (17). The pathophysiology of HF—CS also varies depending on *de novo* vs. acute on chronic HF-CS subtypes. Chronic HF primarily manifests as congestion due to cardiac dysfunction involving increased systemic vascular resistance and redistribution of blood from the splanchnic circulation as previously discussed. These changes, including venoconstriction and elevated CVP, contribute to organ congestion, impaired renal function, and hepatic dysfunction (26). Over time, cardiovascular and muti-organ adaptations to these derangements allow patients with chronic HF to tolerate conditions that would be critically dangerous if they occurred suddenly (86). When ventricular function is severely impaired, chronic HF progresses to HF-CS, resulting in worsening hypoperfusion and subsequent acute on chronic hepatic and renal injury, lactic acidosis, reduced coronary perfusion, and further activation of baroreceptors and chemoreceptors. These factors create a vicious cycle that worsens cardiac function, and the body enters a state of systemic inflammatory response syndrome culminating in multiorgan failure and death.

Initial assessment of HF-CS should include identifying the underlying etiology of HF-CS, the severity of CS using the SCAI stage definitions, and determining the hemodynamic congestive profile. Based on the recent CSWG observational data, 90% of the patients with SCAI B deteriorated to a higher SCAI stage (i.e., C/D/E) during the course of their index hospitalization; the mean time to achieving the maximum SCAI stage was 52 h (36). Hence, early institution of PA catheter guided hemodynamic management may be useful in identifying worsening HF-CS patients sooner and potentially lead to improved outcomes (87, 88). Patients with worsening hypotension and hypoperfusion with elevated lactate levels and evidence of end-organ dysfunction should be transferred to dedicated AHA Level 1 cardiac ICUs with invasive hemodynamic monitoring to reduce the risk of progression to hemometabolic HF-CS. Zweck and colleagues described this phenotype of HF-CS patients with near complete loss of compensatory reflex mechanism to maintain CO combined with multiorgan failure as hemo-metabolic HF-CS, and this subset of patients carry the highest risk of mortality among various HF-CS phenotypes (89).

Elevated filling pressures have been recognized as a strong predictor of adverse outcomes as opposed to CO alone in the advanced HF population (90). Co-administering loop diuretics with thiazides and thiazide-like diuretics (distal sodium reabsorption blockade) or with acetazolamide (proximal sodium reabsorption blockade) can overcome diuretic resistance via sequential nephron blockade and help attain successful decongestion (euvolemia) as shown in the ADVOR (Acetazolamide in Acute Decompensated Heart Failure with Volume Overload) trial, albeit in HF patients without CS (91).

For patients experiencing clinical deterioration and failing to meet hemodynamic goals despite initial therapeutic interventions, a selective and tailored approach to MCS device selection is recommended based on the severity of HF-CS with the goal of achieving hemodynamic unloading and restoring systemic perfusion (59). The application of MCS should be contextualized within a broader strategy aimed at either bridging the patient to advanced therapies or facilitating myocardial recovery. However, it is imperative to exercise caution when considering MCS use if exit strategies are not available; palliative care consultation should be considered if not concurrently pursued. A detailed review of temporary MCS, including IABP, Impella CP and Impella 5.5, and VA ECMO is provided in the sections below.

## CICU management of cardiogenic shock

### Shock teams and regionalized systems of care for CS

Team-based interventions have been shown to expedite care and improve outcomes for a number of high-mortality conditions, such as trauma, cardiac arrest, sepsis and stroke (92–95). The initial experience and feasibility of a team-based approach to CS was first described at the Mayo Clinic in Arizona, where mobile ECMO teams consisting of a cardiac surgeon, perfusionists and critical care nurses were deployed to the community to rapidly resuscitate and stabilize patients in refractory circulatory collapse with VA-ECMO and to transfer them back to local tertiary care center for follow-on care (96). These patients fared better than those stabilized at the community hospital, with survival to discharge of 56% vs. 30%, respectively (96). Given these initial favorable findings and recognizing the time-sensitive clinical nature of CS, researchers issued a clarion call to action in 2015 for centers to employ multidisciplinary “shock teams” in the care of patients with refractory hemodynamic compromise (97). Since then, several dedicated single center shock registries have published their findings following implementation of institutional shock teams, demonstrating associated improvements in short-term outcomes across the severity spectrum and phenoprofiles of CS (Table 1) (11–14). Clinical researchers at Inova Health System were among the first in the country to adopt this care paradigm for patients with both AMI-CS and HF-CS, demonstrating a significant associated improvement in 30-day survival (77% in 2018 vs. 47% in 2016; *p *< 0.001) following implementation of a one-call shock team activation, hemodynamically driven protocols for drug and device-based selection, and institutional best practices around vascular access and closure (11). The Critical Care Cardiology Trials Network (CCCTN), a multicenter collaborative of North American Level 1 CICUs, similarly showed favorable outcomes in 10 out of 24 sites with standardized team-based approached to CS, with significant reductions in CICU mortality (23% vs. 29%; aOR 0.72; 95% CI: 0.55–0.94; *p *= 0.016) and enhanced utilization of PACs and advanced MCS compared to centers without dedicated shock teams (98). Based on the amalgam of this observational data, the 2022 AHA/ACC/HFSA HF guidelines have awarded a class IIa recommendation for the utilization of multidisciplinary shock teams in the triage and follow-up care of patients with CS (99).

**Table 1:** Clinical studies evaluating outcomes in cardiogenic shock after implementation of standardized team-based approach.

Studies	Study design	Number of patients	Shock phenotype	Goals		Interventions	Outcomes	Predictors for mortality
Inova Heart and Vascular Institute Cardiogenic Shock Initiative (11)	Prospective single center (pre- and post-intervention) Compared Shock team vs. historical control	Total = 204 AMI-CS = 81 HF-CS = 122	AMI-CS and HF-CS	a. Rapid CS recognition b. Early MCS c. RHC thresholds after 24h: lactate <3, CPO >0.6W, PAPi >1.0		PCI: 40% RHC: 82% MCS use 66%	a. 30-day survival (*p* < 0.01): 47% (pre-CS team) to 77% at 2 years.	IHVI risk score: 1. Age ≥71 2. DM 3. Dialysis 4. ≥36 h of vasopressor use At 24 h: 5. Lactate ≥3.0 mg/dl 6. CPO <0.6 W 7. PAPi <1.0 Score (30-day mortality) Low: 0–1 (0%), Moderate: 2–4 (18%) High: 5+ (82%)
University of Utah Cardiac Recovery shock team (13)	Prospective single center (pre- and post-intervention) Compared shock team vs. historical control group	Total = 244 AMI-CS = 160 Non-AMI CS = 84	AMI-CS, HF-CS	STEMI a. LVEDP b. LHC +/- PCI c. tMCS consideration d. Urgent RHC	Non-STEMI a. Urgent RHC b. tMCS consideration c. +/- LHC	MCS use: 50% (all shock team patients) - IABP: 30% - Impella: 33% - VA-ECMO: 9% - Combination: 28%	a. Increased in-hospital survival (61% vs. 48%, *p* 0.04) b. Decreased 30-day all-cause mortality (HR 0.61, 95% CI: 0.41–0.93) c. Shock to support time and mean duration of MCS was not significant	AMI-CS, lactate level, and acute kidney injury were independent risk factors of 30-day mortality at the time of MCS initiation
University of Ottawa Heart Institute code shock team (14)	Retrospective single- center (pre- and post-intervention) Compared shock team vs. historical controls	Total = 100 AMI-CS = 13 Non-AMI CS = 87	AMI-CS, HF-CS	a. CS identification and confirmation b. Resuscitation and medical optimization c. tMCS evaluation and initiation d. OHT, LVAD evaluation		PCI: 9% CABG: 1% MCS: 45% (vs. 28% control) PAC monitoring: 62%	a. In-hospital survival 69% vs. 61% (*p* = NS) b. 30-day survival 72% vs. 69% (*p* = NS) c. Increased cumulative survival (HR = 0.53, 95% CI: 0.28–0.99, *p* = 0.03)	–
National Cardiogenic Shock Initiative (12)	Prospective multicenter study Single arm without controls	Total = 171 AMI-CS = 171 Non-AMI CS were not included	AMI-CS only	a. Early identification of CS RHC hemodynamics b. MCS use pre-PCI c. Shock to support time <90 mins d. Ensure TIMI 3 flow e. Complete revascularization f. CPO >0.6 W g. PAPi >0.9		PCI: 100% MCS: 99% - 74% pre-PCI - 7% during PCI - 19% post-PCI RHC: 90%	a. Survival to discharge 72% b. Maintained CPO >0.6 W in 62% c. Door to support time: 85 ± 61 min	Predictors of increased in-hospital mortality 1. Age ≥70 2. Creatinine ≥2 3. Lactate ≥4 4. CPO <0.6
Critical Care Cardiology Trials Network (98)	Prospective multicenter study Compared CICUs with vs. without shock teams	Total = 1,242 - Shock team: 44% - No shock team: 56% AMI-CS = 27% Non-AMI CS = 73%	AMI-CS, HF-CS	a. Rapid identification of CS etiology (AMI vs. non-AMI) and phenotype (LV, RV, BiV) b. tMCS use (type of and total number) c. PAC use d. SOFA score, lactate, and creatinine on CICU admission		Centers with Shock team (vs. without) MCS use: - Overall: 35% (vs. 43%) - Within first 24 h: 60% (vs. 52%) - IABP 58% (vs. 72%) - Impella 28% (vs. 16%) - VA-ECMO 9% (vs. 11%) - PAC use: 60% (vs. 49%)	Center with shock teams (vs. without) a. CICU mortality 23% (vs. 29%, *p* = 0.016) b. Advanced MCS use 53% (vs. 43%, *p* = 0.005) c. New RRT 11% (vs. 19%, *p* < 0.001)	Presence of shock team was independently associated with lower CICU mortality

AMI-CS, acute myocardial infarction complicated by cardiogenic shock; CICU, cardiac intensive care unit; CPO, cardiac power output; CS, cardiogenic shock; DM, diabetes mellitus; HF-CS, heart failure complicated by cardiogenic shock; HR, hazard ratio; IABP, intra-aortic balloon pump; LVEDP, left ventricular end-diastolic pressure; LHC, left heart catheterization; LVAD, left ventricular assist device; MCS, mechanical circulatory support; NS, nonsignificant; OHT, orthotopic heart transplantation; PAC, pulmonary artery catheter; PCI, percutaneous coronary intervention; PAPi, pulmonary arterial pulsatility index; pVAD, percutaneous ventricular assist device; RHC, right heart catheterization; RRT, renal replacement therapy; STEMI, ST-elevation myocardial infarction; VA-ECMO, veno-arterial extra corporeal membrane oxygenation; W, watts.

**Figure 1:**
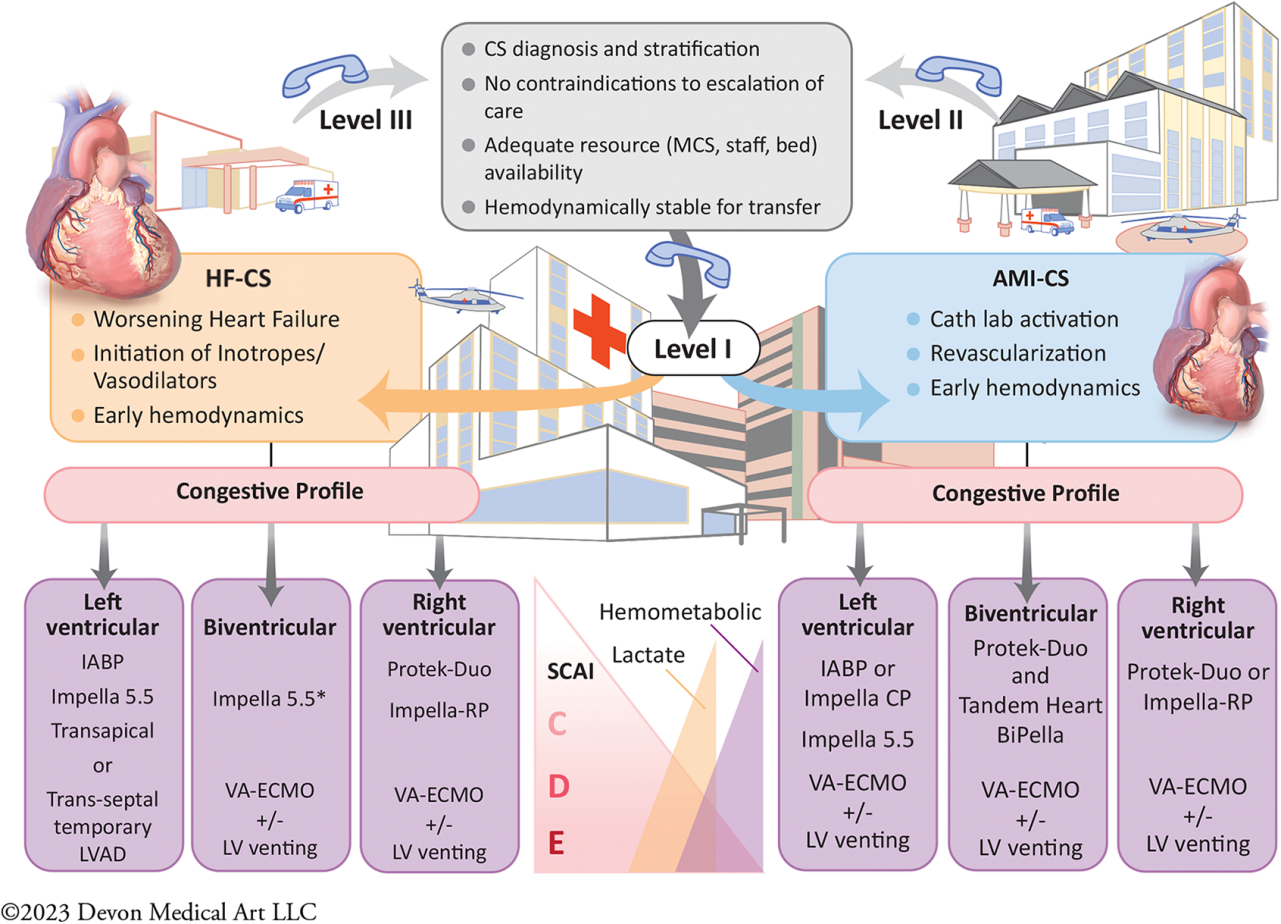
Central illustration. Proposed algorithm of cardiogenic shock management within a regionalized shock network by a multidisciplinary shock team. A contemporary systems of care approach for cardiogenic shock (CS) management by a multidisciplinary team in a “hub and spoke” model. This allows for timely diagnosis with early comprehensive invasive hemodynamic assessment. Early, selective, and tailored mechanical circulatory support (MCS) based on CS phenotype and congestive profiles is crucial for CS management in the modern era. This is also predicated on expedited transfer to the level 1 CS centers of excellence for team-based and comprehensive multiorgan system care. AHF, Advance Heart Failure; AMI, Acute Myocardial Infarction; CS, Cardiogenic Shock; IABP, Intra-Aortic Balloon Pump; LV, Left ventricle; LVAD, Left Ventricular Assist Device; MCS, Mechanical Circulatory Support device, SCAI, Society for Cardiovascular Angiography and Interventions; VA-ECMO, Veno-Arterial Extra Corporeal Membrane Oxygenation.

**Figure 2:**
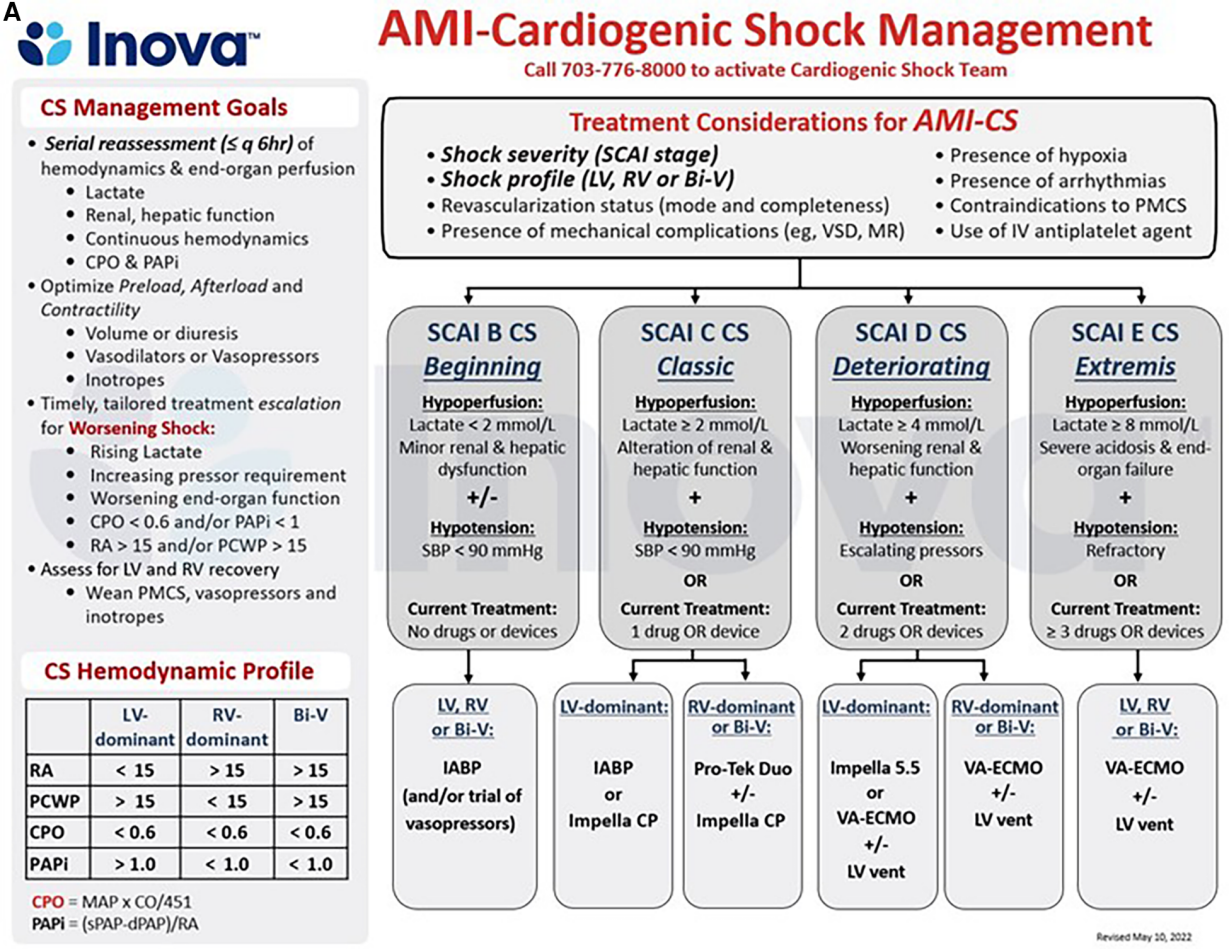
(**A,B**) schematic representation of the care pathways in the upstream and critical care management of patients with acute myocardial infarction (AMI, 4a) and acute decompensated heart failure (HF, 4b) cardiogenic shock (CS) at the INOVA schar heart and vascular institute. BiV, Biventricular; CPO, Cardiac Power Output = [mean arterial pressure x cardiac output]/451; PAPi, Pulmonary Artery Pulsatility Index = [systolic pulmonary arterial pressure—diastolic pulmonary arterial pressure]/right atrial pressure; PMCS, percutaneous Mechanical Circulatory Support; SBP, Systolic Blood Pressure, other abbreviations as in Figures 1, 2.

Addressing regional disparities in management and outcomes remains a major knowledge gap in CS care (59, 100). The 2017 AHA scientific statement on CS endorsed a systems of care approach to management, through the development of regionalized shock networks promoting interhospital collaboration and centralized care at regional destination centers using time-sensitive transfer protocols (1). A tiered-based approach to CS care has been correspondingly described to categorize centers based on the local levels of interventional, surgical, and critical care expertise (55). In these proposed networks, level 1 or “hub” institutions are tertiary or quaternary care centers with 24/7 PCI capabilities, cardiothoracic surgery, and advanced MCS availability. These centers employ “high-intensity” CICUs which are often co-managed by critical care cardiologists and intensivists and provide multiorgan system services and durable cardiac replacement therapies. Level 2 and 3 hospitals, referred to as “spoke” centers, have more limited resources, with the former capable of providing primary PCI services and limited temporary MCS such as IABP, and the latter equipped with emergency medical departments, general medical intensive care units and advanced cardiovascular life support capabilities (Figure 1) (55). Clinical investigators at Inova Health System were among the first in the country to implement and publish an integrated shock network with 34 partnering spoke shock care centers in the Washington DC-Maryland-Virginia region. Through collaboration and integration of care protocols between providers at the spoke institutions and the local shock team at the level 1 center, no significant differences were noted between patients with CS, irrespective of whether they were initially triaged at a Level 1 or 2/3 center, with respect to in-hospital mortality, 30-day mortality, major bleeding complications, stroke, and 30-day major adverse cardiovascular and cerebrovascular complications (101). The 2022 AHA/ACC/HFSA HF guidelines provide a class IIb recommendation for the early triage of patients with CS who are refractory to initial stabilizing measures to level 1 centers with advanced MCS and critical care expertise (99). As we continue to learn about the advancements in the field of cardiogenic shock and its implications on patient management with use of tMCS devices, the original protocol for management of CS put forth by our task force at Inova has undergone several revisions and the most recent formulation stratified by AMI-CS and HF-CS are presented in Figures 2A,B, respectively. The CS team activation and coordination protocols are presented in Supplementary Figures 1 and 2.

**Figure:**
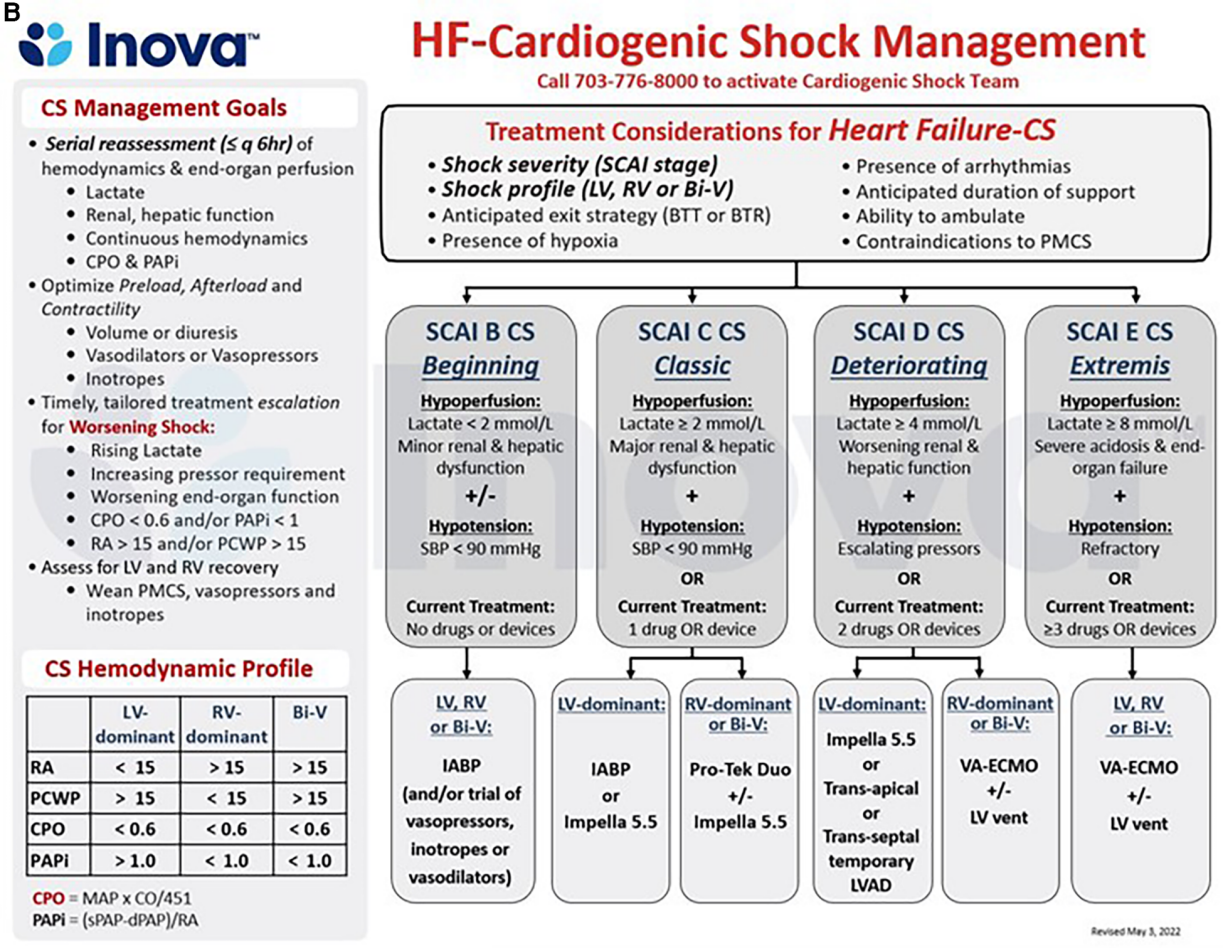


### Vasopressors and inotropes

The acute management of patients with CS requires successful augmentation of cardiac output, typically with intravenous vasopressors and inotropes (102). These agents may enhance myocardial contractility by targeting the system of myocardial calcium fluxes, modulating adrenergic receptors or through phosphodiesterase inhibition (102). Although norepinephrine is often recommended as the first-line agent of choice for treatment of AMI-CS, data from clinical trials is limited. A subgroup analysis of the SOAP-II trial showed that norepinephrine appeared superior to dopamine based on fewer deaths in the CS subgroup (103). Epinephrine was associated with higher incidence of refractory AMI-CS than norepinephrine in the OptimaCC Study (56). Milrinone was associated with greater rates of sustained hypotension and atrial arrhythmias without any difference in cardiovascular-related hospital days as compared to placebo in the OPTIME-CHF Study (104). The DOREMI (Dobutamine Compared with Milrinone) trial, a double-blind randomized clinical study comparing dobutamine with milrinone in patients with AMI and HF-related CS, did not find any differences with regard to the composite or individual end-points of in-hospital death, resuscitated cardiac arrest, non-fatal myocardial infarction, transient ischemic attack, stroke, renal replacement therapy or need for mechanical circulatory support or heart transplant between patients randomized to milrinone or dobutamine (6). Despite frequent and prolonged administration of these drugs in the treatment of patients with CS, they all may have deleterious effects on energetic efficiency, as they may further increase myocardial oxygen demand by way of refractory ischemia, arrhythmias, and vasodilatory hypotension (102). An observational analysis from the CSWG registry demonstrated that concatenation of vasoactive therapies, independent of tMCS, was associated with worse mortality. Given that multiple vasoactive and/inotropic agents are often used contemporaneously, concerted efforts should be made to properly assess the drug(s) of choice based on their effects on right and/or left ventricular congestive profiles and to minimize the total number and duration of administration of vasopressors and inotropes (59). Clinical guidelines currently endorse a Class I recommendation for the utilization of vasopressors and/or inotropes in CS in the 2022 AHA/ACC/HFSA Guidelines for Heart Failure (105).

### Invasive hemodynamic monitoring

A growing body of evidence now supports routine early invasive hemodynamic assessment with PAC use for HF-CS despite initial preliminary studies failing to show benefit (11). The Evaluation Study of Congestive Heart Failure and Pulmonary Artery Catheterization Effectiveness (ESCAPE) trial, a randomized controlled trial (RCT) of 433 patients with symptomatic, recurrent HF without CS who were assigned to PAC-guided volume optimization or clinical assessment alone failed to demonstrate an impact of PAC use on primary composite endpoint of days alive and out of the hospital at 6 months (106). Most notably, however, the ESCAPE trial did not specifically evaluate patients with CS for whom inotropic and vasopressor therapy was frequently used or for assessment of potential candidacy for MCS (107). The eligibility criteria in the ESCAPE trial also excluded patients with severe HF decompensation, significant renal dysfunction (creatinine >3.5 mg/dl), prior use of inotropic therapy, and those with pulmonary hypertension—a subset of patients who ultimately may have benefitted most from PAC-guided therapy (107). Nevertheless, the overall use of PAC among patients with HF continued to decline from 2004 to 2014 including in patients with CS (88).

Subsequently, given the controversy and limitations of ESCAPE, there have been several registries and emerging studies advocating for the incorporation of early invasive hemodynamic monitoring as a standard of care in contemporary CS management, as PAC use may lead to earlier and more accurate identification of CS phenotyping to tailor specific medical and device-based therapies (11). A contemporary analysis of 1,531,878 CS admissions from the NIS database from 2004 to 2018 observed significant regional variations in the employment of invasive hemodynamics, with the highest rate of PAC use in the Northeast United States and in urban teaching hospitals (22). Based on these findings and subsequent negative studies, societal guidelines have provided only limited recommendations regarding the use of PACs in CS, with the 2022 AHA/ACC/HFSA guidelines for the management of HF providing a Class IIa recommendation for invasive hemodynamics only in select patients with HF and worsening symptoms or signs of end organ perfusion despite optimal medical therapy and a Class IIb recommendation for its use in CS (99).

There are several potential theoretical advantages to the routine use of upfront, routine invasive hemodynamics in CS which have been postulated: (1) Timely diagnosis of CS, including assessment of RV and LV congestive profiles and elucidation of SCAI classification; (2) Optimal use of hemodynamically tailored tMCS with serial follow-up assessments to inform decision making around device escalation or de-escalation; and (3) Correlation of cardiac performance with congestion to further inform risk stratification of patients with CS and multiorgan system dysfunction (33, 36). There is an emerging body of evidence suggesting that routine PAC use may be associated with improved survival in CS, particularly in patients with tMCS (11, 12, 88, 108, 109). These observational contemporary studies have demonstrated reductions in CICU- and in-hospital mortality in patients with AMI-CS and HF-CS across all SCAI stages, particularly when complete PAC data was obtained within 6 h of hospital admission and prior to tMCS implantation (88, 108). Furthermore, routine invasive hemodynamic monitoring has been a core element in contemporary CS treatment algorithms (33). For instance, the National Cardiogenic Shock Initiative (NCIS), a single-arm, prospective, multicenter registry of AMI-CS in the United States, found that using a standardized protocol emphasizing routine invasive hemodynamic monitoring and rapid initiation of tMCS resulted in a 72% survival to discharge rate for patients presenting with AMI-CS (12). This was indeed achieved through an aggressive utilization of PAC (performed in 92% cases) and tMCS implanted in 74% prior to PCI. Likewise, recent outcome data analysis from the CSWG registry demonstrated that the mortality rates differed significantly between patient groups with no PAC profiling, incomplete PAC profiling, and complete PAC profiling (26). Patients in the complete PAC assessment group had the lowest in-hospital mortality rates across all SCAI stages [aOR 1.57 (95% CI 1.06–2.33), compared with no PAC assessment] (43). Furthermore, in an analysis of administrative data from the Nationwide Readmissions Database including over 230,000 hospitalizations for CS between 2016 and 2017, the use of PAC was associated with a significantly lower in-hospital mortality [aOR 0.69 (95% CI 0.66–0.72)] (110). More recent data from the CCCTN, a multicenter network of level I CICUs in the United States across 34 centers, again demonstrated that PAC use, even after adjustment for factors associated with their placement, was associated with lower mortality in all shock patients admitted to a CICU [OR 0.79 (95% CI 0.66–0.96); *P* = 0.017] (88). Currently, the latest European Society of Cardiology Guidelines for Acute and Chronic Heart Failure have recommended that invasive hemodynamic monitoring coupled with a standardized team-based approach may be associated with improved patient survival (111). An expert consensus statement from SCAI also stressed that defining CS phenotypes using invasive hemodynamics may help to guide therapy, particularly with respect to identifying patients who may require RV or BiV support (35).

Given that no RCTs have prospectively tested the utility of PAC use among patients with acute decompensated HF-CS (ADHF-CS), the Pulmonary Artery Catheter in Cardiogenic Shock (PACCS) trial (NCT05485376) is a registry-based trial designed by the CSWG and will test the hypothesis that early invasive hemodynamic assessment (within 6 h of randomization) and ongoing management with a PAC decreases the primary endpoint of in-hospital mortality compared to clinical management with delayed (beyond 48 h after randomization) or no PAC-guided assessment among patients with ADHF-CS (112). As a randomized, multicenter, adaptive design trial, PACCS intends to enroll 400 patients across several sites in the United States with a study duration of 4 years (112). Outside of hemodynamic monitoring in the inpatient setting, there remains a paucity of data testing the efficacy of remote hemodynamic monitoring in the outpatient setting to prevent adverse outcomes following CS hospitalization. The Hemodynamic monitoring to prevent Adverse events foLlowing cardiOgenic Shock (HALO Shock) trial (NCT04419480) is a prospective, unblinded study at the Inova Schar Heart and Vascular Institute that will test the hypothesis in a 1:1 randomized fashion that remote hemodynamic monitoring with CardioMEMS (Abbott) device implementation improves outpatient management including medication titration to prevent adverse outcomes following CS hospitalization (113). HALO Shock is a pilot study that has the potential to transform care for shock survivors with persistent congestion at high risk of subsequent morbidity and mortality following CS.

### Temporary mechanical circulatory support devices

Temporary mechanical circulatory support (tMCS) devices are increasingly utilized in the management of CS worldwide. An analysis of 110,462 AMI-CS admissions from the NIS database from 2005 to 2014 demonstrated that tMCS was used in 55% of cases (114). Similar findings have been noted in European registries, yet with shifts toward greater employment of advanced tMCS platforms such as microaxial flow percutaneous ventricular assist devices (pVAD) and veno-arterial extracorporeal membrane oxygenation (VA-ECMO) (115). To date, RCTs evaluating pVAD and VA-ECMO in AMI-CS have not demonstrated an improvement in short-term outcomes compared to either medical therapy or conventional IABP counterpulsation (3–5). Recent large scale administrative claims data studying the use of pVADs in AMI-CS, in fact, suggest that routine employment of these devices in the management of CS patients may be associated not only with increased risk for short-term and one-year mortality, but also major bleeding complications, acute kidney injury, and stroke (66, 116, 117). As a result, the indications for tMCS in AMI-CS, timing of implantation, and how to best incorporate them into shock management protocols remains an evolving area of controversy, and clinical practice guidelines currently delineate the routine employment of these devices in AMI-CS care as a Class IIb (Level of Evidence: C) recommendation (44, 117). Mechanistically, tMCS devices may serve as bridging vehicles to myocardial recovery or cardiac replacement therapies by not only reducing intracardiac filling pressures and LV stroke work but also augmenting coronary and end-organ perfusion (59). Optimal tMCS device selection should be tailored to not only severity of the shock state, but also the respective congestive profiles and phenotypes, thus requiring a nuanced understanding of how each device platform can modulate right and left ventricular pressure-volume loop hemodynamics (26, 59). Given regional variations in the expertise and management of tMCS, centers caring for patients with these devices should employ standardized protocols not only around device insertion and removal, but also continuous monitoring in the CICU to mitigate the associated risks for bleeding, vascular complications, hemolysis, and afterload mismatch associated with these devices (68, 118). Ongoing trials evaluating the role of tMCS devices in CS are summarized in Table 2.

**Table 2:** Ongoing, select clinical trials assessing the role of mechanical circulatory support devices.

Trial	Number of patients	Study type	Intervention	Control	Primary outcome
REVERSE (NCT03431467)	96	Multicenter RCT	Impella-CP LV Vent + VA-ECMO	VA-ECMO	30-day survival free from MCS, OHT, or inotropic therapy
DanGer Shock (NCT01633502)	360	Multicenter RCT	Impella CP ± Inotropes	Conventional circulatory support	All-cause mortality
Altshock-2 (NCT04369573)	200	Multicenter RCT	Early IABP (within 6 h)	SoC (vasopressors/Inotropes)	60-day survival or successful bridge to durable LVAD or OHT
JENAMACS (NCT04451798)	20	Single center prospective study	Impella CP	–	Acute hemodynamic effects measured by PAC and echocardiographic evaluation of BiV function
UNLOAD-AMI (NCT04562272)	80	Single center RCT	Impella CP for 36–48 h + SoC after PCI	SoC	Difference in LVESV, extent of post-infarct scar
SMART-RESCUE II (NCT04143893)	1,000	Multicenter prospective observational study	tMCS + medical management	Optimal medical management	All-cause mortality at 3 months
RECOVER IV (NCT05506449)	560	Multicenter RCT	Impella CP prior to PCI in STEMI + PAC	Medical management ± IABP	30-day all-cause mortality
ALLOASSIST (NCT03528291)	240	Prospective multicenter observational study	Transient circulatory support (VA-ECMO, Impella)	SoC	In-hospital mortality from inclusion day to 6 months
UNLOAD-ECMO (NCT05577195)	198	Multicenter RCT	Impella + VA-ECMO	VA-ECMO	Time to death from any cause within 30 days

LVESV, left ventricle end-systolic volume; RCT, randomized clinical trial, SoC, standard of care; other as described in preceding figures and tables.

**Figure 3:**
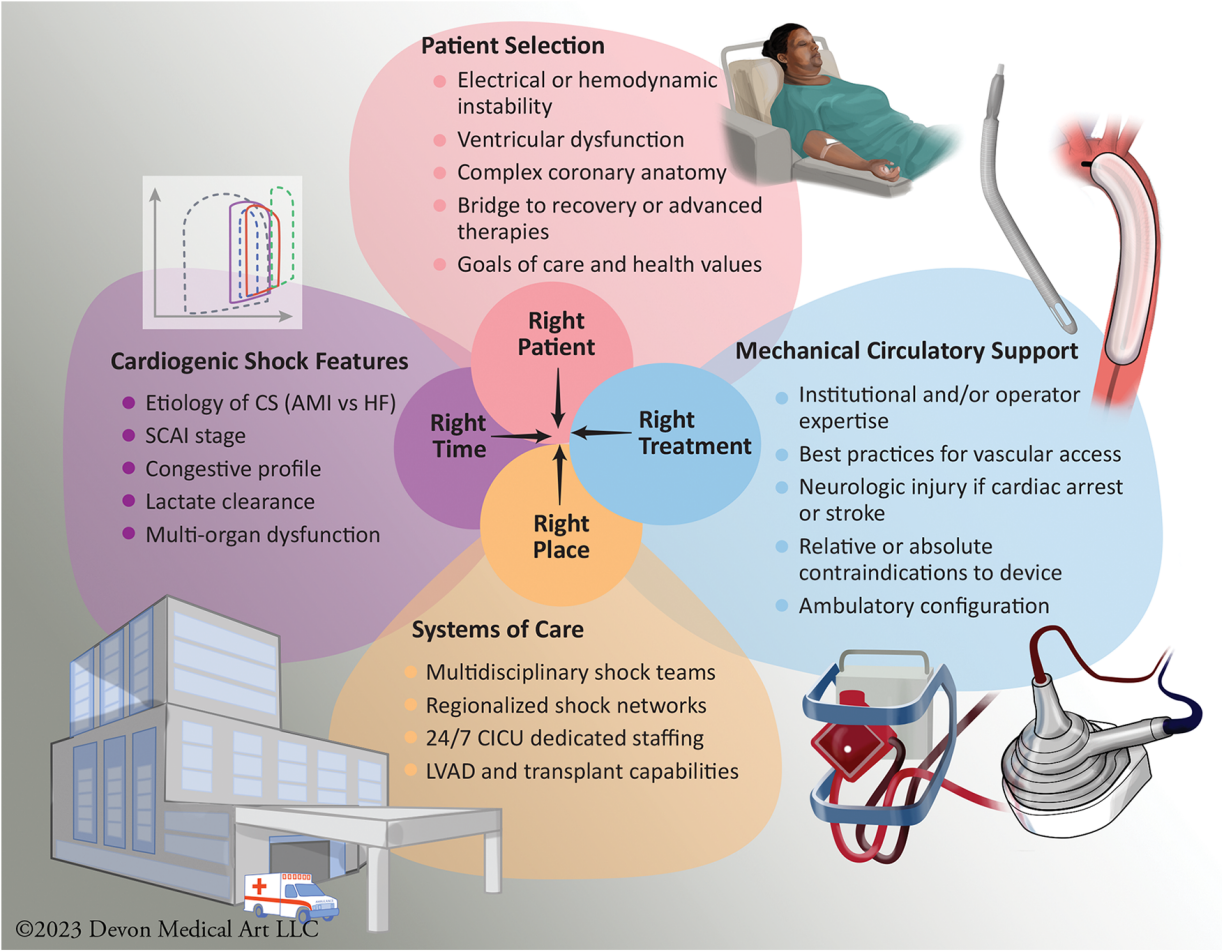
Optimizing patient-centric care: mechanical circulatory support considerations for appropriate use of selective and tailored approach to available devices. The figure illustrates the intricate process of achieving optimal patient-centered care in the context of MCS use. The achievement of the right patient, at the right time, with the appropriate MCS device, and in the right clinical setting who should be managed at an appropriate level of CS center in a regionalized shock network, is a complex endeavor influenced by a multitude of factors. AMI, Acute Myocardial Infarction; CICU, Cardiac Intensive Care Unit; CS, Cardiogenic Shock; HF, Heart Failure; LVAD, Left Ventricular Assist Device; SCAI, Society for Cardiovascular Angiography and Interventions.

There is emerging data from several North American CS single-center registries that select utilization of tMCS devices based on time-sensitive protocols may be associated with improved survival (12, 119, 120). The 2022 AHA scientific statement on tMCS similarly endorsed an algorithmic approach to device selection and subsequent escalation and de-escalation of tMCS using a standardized framework which integrates timely, interdisciplinary input and collaboration based on serial invasive hemodynamics, clinical, and laboratory data (121). While early implantation of tMCS device to offset or reverse the ensuing end-organ dysfunction may seem attractive, the deleterious risks associated with these devices and their potential complications needs to be appreciated given the absence of any robust RCT supporting mortality benefit from instituting tMCS (Figure 3). In select patients with increasing number and doses of inotropes and/or vasopressors, one must consider escalation to tMCS strategy in order to minimize the risk of arrhythmias and increasing myocardial oxygen demand. An interdisciplinary shock team evaluation as soon as CS is identified is of utmost importance, so that appropriate and tailored MCS device can be deployed. Although no absolute level of lactate reliably differentiates between patients with and without poor prognosis, early lactate clearance within the first 6–8 h of CS onset and/or at 24 h has shown to be more prognostic in identifying treatment responders and associated with overall improved survival (32, 122). The International Society for Heart and Lung Transplantation, in collaboration with the Heart Failure Society of America, has published guidelines on employing tMCS in specific populations, including women, ACHD, the elderly and frail, as well as those who are obese. These guidelines address implementation timing, patient specific device selection, and emphasizes the importance of involving patients and their families in the decision-making process through the use of decision aids (123).

### Intra-aortic balloon pump

IABP plays a crucial role in cardiac support by employing the principle of counterpulsation. These medical devices consist of balloon-mounted catheters with a volume capacity ranging from 25 to 55 cc, strategically positioned in the descending aorta. The mechanism involves inflation during diastole, which enhances central aortic root diastolic pressure and coronary perfusion. During systole, the balloon deflates, creating a negative pressure zone, consequently reducing LV afterload. This leads to a decrease in LV cardiac work and myocardial oxygen consumption while simultaneously augmenting cardiac output by up to 1 L/min. The clinical efficacy of IABPs has been explored through randomized controlled studies, almost exclusively in cases of AMI-CS (124, 125). The IABP-SHOCK II trial randomized AMI-CS patients to IABP or standard therapy and found no significant difference in 30-day mortality, leading to the conclusion that routine IABP use did not improve survival in this patient population (124). In contrast, IABPs have been associated with improved outcomes in patients with HF-CS, albeit in nonrandomized studies, serving as a bridge to durable LVAD or orthotopic heart transplantation (HT) (126–129). Notably, there is some evidence to suggest the presence of IABP-responders and non-responders. IABP insertion has shown benefits in patients with non-ischemic cardiomyopathy and higher pulmonary artery pulsatility index (PAPi) scores, indicating its potential utility in appropriate patient selection for HF-CS (126). High SVR, low cardiac index at baseline, and diabetes mellitus have also been shown to be positive predictors of IABP response (130). The ongoing AltShock-2 trial (NCT04369573) aims to address this knowledge gap by prospectively comparing IABP to vasoactive therapy in HF-CS patients, with a primary endpoint of 60-day survival or successful bridge to OHT (131).

### Percutaneous ventricular assist device (pVAD)

Impella pVADs, developed by Abiomed Inc. (Danvers, MA), are catheter-based microaxial ventricular assist devices that can deliver up to 5.5 liters of cardiac output by using the principle of an Archimedes screw, harnessing the impeller’s rotational kinetic energy to displace blood from LV to aorta, thereby reducing the LV preload and oxygen consumption while increasing mean arterial pressure and tissue perfusion (132). In the U.S., Impella devices have been FDA approved to provide temporary support for 5–7 days (or 30 days for Impella 5.5), regardless of heart rate or residual LV contractility. However, limited randomized data on the efficacy of Impella on survival in CS have led to lack of endorsement from professional society guidelines, potentially causing variations in device selection and use across centers. Studies like ISAR-SHOCK and IMPRESS-in-Severe-SHOCK have compared Impella to IABP in AMI-CS (4, 132). While these studies have shown improvements in cardiac index with Impella, especially when initiated before primary PCI, they have not demonstrated reduced 30-day mortality as compared to IABP. Additionally, a meta-analysis involving percutaneous MCS devices has reported no significant mortality difference between Impella and IABP (133). However, it highlighted a higher bleeding risk associated with tMCS (134). RECOVER IV is a prospective, multicenter, randomized controlled open-label, two-arm trial with an adaptive design evaluating whether early Impella support in STEMI patients with CS prior to percutaneous intervention as compared to a non-Impella based standard of care treatment strategy reduces all-cause mortality at 30 days. Emerging interest surrounds pre-procedural LV unloading to enhance coronary reperfusion and clinical outcomes, with ongoing randomized trials investigating this novel therapeutic approach (135, 136). Impella RP Flex is a newer device specifically designed for right ventricular (RV) support, with an inlet either in the inferior or superior vena cava and an outlet in the pulmonary artery. When combined with left-sided Impella pumps, comprehensive biventricular support may be provided although this strategy has not been formally tested in clinical trials. Impella RP, the immediate predecessor of the Impella RP Flex, has shown safety and immediate hemodynamic benefits in the RECOVER RIGHT trial (137). In summary, the Impella platform of devices offer significant hemodynamic support in CS, particularly in AMI-CS cases but without any randomized data evidence of survival benefit to date. Thus, there is a need for more comprehensive clinical data to identify the appropriate patient population for timely device selection to optimize outcomes and inform clinical practice guidelines.

TandemHeart (LivaNova, Houston, TX) is percutaneously placed via a transseptal puncture for left sided support (TH-LVAD) and with ProtekDuo, supports the right side as well (TH-RVAD) (138, 139). TH can be utilized in cases such as LV thrombus and/or significant aortic regurgitation where transvalvular options are limited. By splicing an oxygenator into the circuit, it facilitates hemodynamic stabilization, perfusion, and decongestion with oxygenation analogously to VA-ECMO. Given the transseptal approach, the placement of TH-LVAD is complicated in the emergent setting and no mortality benefit has been proven for these devices yet (140, 141).

### Extracorporeal membrane oxygenation (ECMO)

ECMO is commonly used as the first line tMCS strategy in patients with acute severe or refractory cardiac and respiratory failure. The configuration of ECMO determines whether it is providing gas exchange (veno-venous or VV-ECMO) or gas exchange with hemodynamic support (VA-ECMO) (142, 143). ECMO support can be inserted percutaneously via a large bore venous cannula inserted in a central vein which drains the de-oxygenated blood, cycles through an external oxygenator and a blood pump (centrifugal or rotational), and returns the oxygenated blood to a central artery via large bore arterial cannula. To prevent limb ischemia, a distal perfusion cannula is often used, usually placed through the superficial femoral artery and sometimes through the posterior tibial artery in a retrograde fashion. Typical access sites for VA ECMO cannulation include femoral or axillary arteries and jugular or subclavian veins (144–146). A key limitation to prolonged support with VA-ECMO is increased LV afterload which will be discussed in detail in the subsequent section. Systemic anticoagulation is needed because of the large cannula size, which exposes patients to the possibility of multiple complications; overall ECMO-associated morbidity and mortality are high. A 2014 meta-analysis of almost 1,900 patients reported up to 20% rate of lower extremity ischemia, up to 50% acute renal failure needing dialysis, and up to 40% rate of major bleeding in patients supported with VA-ECMO for CS and cardiac arrest (147). The ECMO-CS trial was recently published, comparing early VA-ECMO vs. salvage VA-ECMO in 122 patients with CS SCAI stage D or E (5). The trial enrolled around two-thirds AMI-CS patients excluding patients who were comatose after cardiac arrest. Unfortunately, the trial failed to demonstrate any significant difference between the two groups in the 30-day composite primary end point of death, resuscitated cardiac arrest, or escalation of MCS (63.8% vs. 71.2% respectively; hazard ratio, 0.72; 95% CI: 0.46–1.12); 30-day mortality did not differ (50.0% vs. 47.5%) (5). The trial had comparable but high rates of serious adverse events (60.3% vs. 61.0%). In addition, the multicenter, international EURO SHOCK trial aimed to determine if early use of VA-ECMO within 6 h (± only IABP for unloading) improves 30-day mortality in patients with persistent CS 30 min after primary PCI as compared to standard of care therapy (148). The trial aimed to enroll 428 patients but was able to enlist only 35 (13.25%) patients due to recruitment challenges amidst the coronavirus disease (COVID-19) pandemic. Patients with AMI complications such as ventricular septal rupture, ischemic mitral regurgitation, or LV free-wall rupture were excluded. The trial failed to demonstrate a significant difference in all-cause mortality at 30 days between the two groups (43.8% vs. 61.1%, HR = 0.56, 95% CI: 0.21–1.45, *p* = 0.22). The secondary outcome of major bleeding and vascular complications were numerically higher in the VA-ECMO group. Mortality at 12-months was numerically lower but statistically not significant in the VA-ECMO group (51.8% vs. 81.5%, HR = 0.52, 95% CI = 0.21–1.26, *p* = 0.14) (148). Given the low recruitment and limited patient enrollment, no definitive conclusions can be drawn from this trial. Most recently, the ECLS-SHOCK (ExtraCorporeal Life Support for Acute Myocardial Infarction complicated by Cardiogenic SHOCK) trial enrolled 420 patients with AMI-CS and randomized to ECLS or usual care group and reported no significant 30-day mortality difference (47.8% vs. 49.0%, *p* = 0.81). ECLS was associated with increased incidence of moderate or severe bleeding (23.4% vs. 9.6%, *p* < 0.05) (8). In light of the aforementioned studies, the optimal role of VA ECMO in AMI-CS remains to be determined.

### LV venting

**Figure 4:**
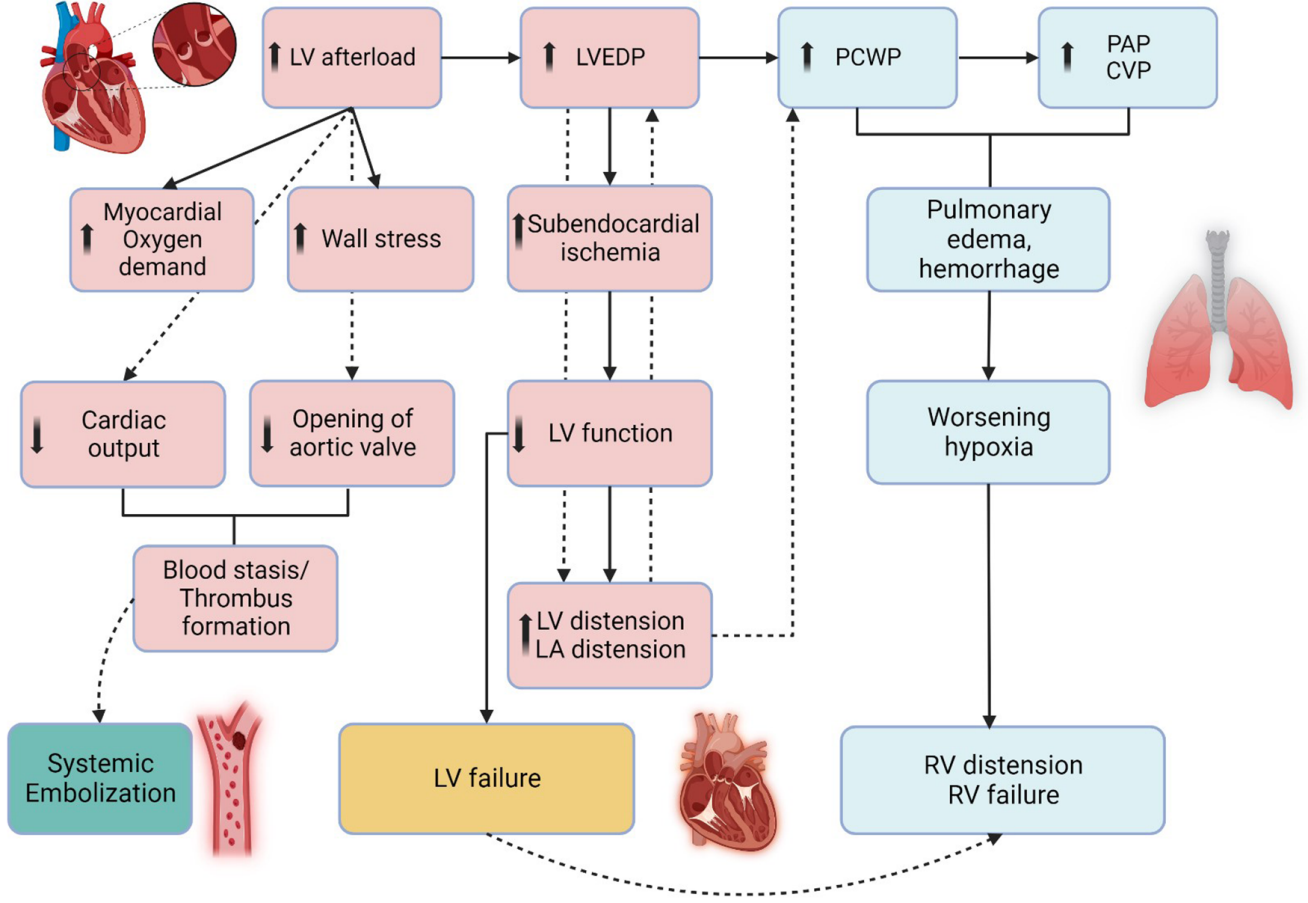
Pathophysiology of left ventricular distension during veno-arterial extracorporeal membrane oxygenation. The figure illustrates the pathophysiology of left ventricle (LV) distension which occurs during VA-ECMO as the outflow cannula generates retrograde flow towards the aortic valve, resulting in increased afterload. This results in high LV end-diastolic pressure (LVEDP) and increased LV end-diastolic volume (LVEDV), resulting in subendocardial ischemia, hindering LV recovery. In some cases, mitral valve may act as “pop-off” valve for the LV but leads to pulmonary edema, resulting in increased PCWP, CVP, and subsequently RV distension and RV failure. LV, CVP, Central Venous Pressure; EDP, End-Diastolic Pressure; LV, Left Ventricle; PAP, Pulmonary Artery Pressure; PCWP, Pulmonary Capillary Wedge Pressure, RV, Right Ventricle. Created with BioRender.com.

During peripheral VA-ECMO, retrograde flow generated towards the aortic valve by the arterial cannula increases LV afterload (149). The increased afterload produces LV distension increasing wall stress and oxygen demand of the myocardium (149, 150). Elevated LV end-diastolic pressure, worsens subendocardial ischemia thereby further reducing LV systolic function (Figure 4). In AMI-CS, with competent mitral valve and non-compliant LV, the risk for LV distension is higher as compared to patients with HF-CS (i.e., in chronic decompensated HF, the LV is often dilated with mitral annular dilatation), in which the mitral valve serves as a potential “pop-off” valve and leads to acute pulmonary edema. In AMI-CS cases supported with VA-ECMO recovery can be delayed and often difficult owing to the above mechanisms. An artificially high afterload results in the inability of the aortic valve to open, LV blood stasis, and thrombus formation with the potential risk of embolization to systemic circulation. The effect of increasing LV end-diastolic pressure results in elevated LA pressures, which in turn increases pulmonary venous pressure, resulting in pulmonary edema, hemorrhage, and eventually systemic hypoxia (151). Multiple strategies for LV venting and/or unloading can be used and each have their own advantages and disadvantages (Table 3). Common practices include reducing the ECMO flow, increasing inotropic therapy, or left ventricular venting via a pVAD insertion, left atrial septostomy, and surgical venting (Table 3).

**Table 3:** Strategies for left ventricular unloading during veno-arterial extracorporeal membrane oxygenation.

Strategy	Advantage	Disadvantage
1. Augment Inotropes	• Increases LV contractility • Enhance AV opening • May decrease afterload	• Increase myocardial oxygen demand • Increase arrhythmogenic events • Increases LV work
2. Reducing ECMO flow	• Reduces LV loading • Increases AV opening	• Decreases cardiopulmonary support
3. IABP	• Increases coronary blood flow • Decreases afterload • Can be placed at bedside • Relatively inexpensive • Can be used if LV thrombus +	• No direct LV unloading • Survival benefits questionable • Risk of thrombosis, access site bleeding, atheroemboli • Contraindicated in AI • Renal failure/mesenteric ischemia due to malposition
4. Impella (Impella CP, 2.5, 5.5)	• LV decompression • Provides antegrade flow	• Hemolysis • Vascular/limb injury • Expensive • Contraindicated if LV thrombus +/moderate to severe AI/PAD
5. Surgical (Trans-apical, trans-pulmonary, Trans-septal)	• Reliable and strong unloading	• Invasive • Increased complications risk • Limited to post-cardiotomy shock

AI, aortic insufficiency; AV, aortic valve; LV, left ventricle; PAD, peripheral arterial disease.

Peripheral cannulation coupled with an LV vent using either an intra-aortic balloon pump for passive venting or Impella CP/5.5 for active unloading is a frequently employed strategy. Furthermore, surgical assist devices such as the CentriMag pump can provide full univentricular or biventricular support, approximately 5 liters per minute, for extended periods. This approach involves venting the left ventricle through an inflow cannula in the left atrium or the LV apex with an outflow cannula into the aorta (152). For right ventricular support, an inflow cannula is placed in the right atrium with an outflow cannula in the pulmonary artery. This strategy is typically reserved for patients inadequately supported by percutaneous assist devices who are awaiting heart transplantation, anticipating prolonged wait times.

### Anticoagulation and anti-thrombotic therapy in temporary mechanical circulatory support devices

The use of tMCS is associated with both bleeding and thrombotic complications, which may occur simultaneously in some patients, which may pose a challenge in finding an optimal anticoagulation and anti-thrombotic regimen. In devices such as VA-ECMO, IABP, and Impella (Abiomed Inc), use of anticoagulation is mandatory to counteract activation of the coagulation cascade caused by shear force stress and the presence of a foreign material in the body, in order to prevent device-related thrombosis and embolization. This is mostly based on pathophysiological considerations as opposed to randomized clinical trial evidence, because such trials are lacking. While on the other hand, recent large retrospective studies including patients supported by microaxial flow pumps, showed higher mortality as compared to those supported by IABP, mainly due to higher rates of major bleeding complications (66, 116). Apart form use of anticoagulation, the effect of DAPT in patients with acute coronary syndrome post PCI, or those who develop acquire von Willebrand syndrome, or vascular access-site complications, the risk of bleeding increases even further. In general, unfractionated heparin (UFH) is the anticoagulant agent of choice in patients on pVAD (153, 154). In subset of patients who develop heparin-induced thrombocytopenia (HIT), use of direct thrombin inhibitors (DTIs), such as bivalirudin or argatroban, is recommended (155, 156). When used, UFH has marked variability in its anticoagulation effect in different patients and monitoring can be challenging. Also, due to presence of comorbidities and multi-organ dysfunction in these critically ill patients, aPTT may not provide an accurate assessment of its anticoagulant effect. Therefore, parallel monitoring of anti-Xa and aPTT is considered to be superior to aPTT alone because it is not affected by other confounding factors, and has been supported by studies that show mortality increases when aPTT and anti-Xa start to diverge (154, 157–159).

### Long term outcomes for survivors of cardiogenic shock

Intermediate and long-term outcomes in CS patients has become an important area of investigation. The CSWG demonstrated that in-hospital mortality was significantly higher in patients with AMI-CS (39.5%) as compared to HF-CS (25.3%; *P* < 0.0001) despite having similar hemodynamic profiles (43). In a Nationwide Readmission Database study, there was a 16% readmission rate among 4,229 survivors of CS post-ECMO who recovered and were discharged alive (160). These patients had an in-hospital mortality rate of 10% with the most common cause of re-admission being infection followed by acute decompensated heart failure (160). Additional CS studies analyzing patients with AMI-CS and non-AMI-CS (not restricted to ECMO use), respectively, reported a higher readmission rate of 20% and 23%, respectively (161, 162). In a more recent study by our group, CS patients who survived to hospital discharge had comparable 30-day readmission rate for HF-CS and AMI-CS (19.5% vs. 24.5%; *p* = 0.30) (17). Our group reported similar outcomes with higher in-hospital mortality for AMI-CS when compared to HF-CS (39% vs. 24%, *p* < 0.001); however, in patients who survived to discharge, the one-year mortality was comparable for both groups (19.7% vs. 23.5%, *p* = 0.41) (17). However, limited data exists on the long-term outcomes, beyond the first year following index hospitalization of patients surviving the initial phase of CS. In the 2005 French registry of Acute ST-elevation and non-ST-elevation Myocardial Infarction (FAST-MI), 59% of patients with CS were alive at 5 years with increased risk of death in the first year after discharge (163). A recent population-based retrospective Canadian study of 9,789 AMI-CS patients showed that one-year mortality was 41% and at 5 years was around 60% (164). Among patients who survived to discharge, 48% were re-admitted to the hospital and 15% died within the first year, suggesting potential opportunities to serve CS survivors during this vulnerable phase of their illness journey (164).

### Role of palliative and hospice care in CS management

Despite the wide availability of pharmacological and device-based therapies, not all CS patients may benefit from them, and the illness culminates in death for many patients (165). Palliative care services can provide social, emotional, and spiritual support to CS patients and their families and is distinguished from hospice care with its focus on controlling symptoms and improving quality of life concurrently while complementing curative therapies (1, 166, 167). Feng at al. analyzed 2017 Nationwide Readmission Database with 134,000 CS admissions with a reported mortality of 36% (168). Only 9% of CS admissions utilized palliative care services which was associated with lower 30-day readmission rate of 12% as compared to 22% in those who did not see palliative care. The hospitalization cost per patient was also lower at US $51,000 vs. $67,000, findings showing benefit not only for the individuals but also for health care systems across the US (168). Unfortunately, observational data suggest patients receiving palliative care in AMI-CS is very low at 5% in the last 15 years but there is an increasing trend toward more frequent utilization of these services (169).

### Knowledge gaps and future directions for CS

There are several important knowledge gaps which provide fertile opportunities for future investigation in CS including but not limited to: electronic health alerts for early identification and recognition of CS; clinically actionable risk prediction tools; “Shock-omics” phenotyping and machine learning approaches to CS; pragmatic, registry based RCTs in CS; and longitudinal survivorship in CS.

### Role for early identification

CS is a heterogeneous syndrome with protean clinical manifestations. Rapid, early identification and stratification are of utmost importance in management of CS. The CSWG observational data indicate that 90% of SCAI B and 70% of SCAI C CS patients, respectively, progress to a higher SCAI stage during the course of their index hospitalization (36). This is particularly important as SCAI stage at admission was associated with higher in-hospital mortality, which further increased after re-classifying them at 24 h in the Altshock-2 registry (SCAI B = 18%, SCAI C = 27%, SCAI D = 63%, SCAI E = 100%), and was also an independent predictor of in-hospital mortality (170). Electronic health alerts built within the electronic medical record (EMR) interface may be useful in early identification analogous to the “sepsis alert” which is based on SIRS or Sequential Organ Failure Assessment (SOFA) criteria. Application of “cardiogenic shock alert” can be considered by utilizing SCAI stages, clinical history, demographics, and hemo-metabolic markers of organ perfusion (such as lactate, renal function, transaminase levels, SBP, and/or MAP). This may potentially expedite timely diagnosis and management and transfer to a higher level of care when appropriate. However, “alert fatigue” is a common phenomenon in which clinicians often ignore EMR safety notification at a rate between 49% and 96% (171). False alerts for non-shock patients are a major cause of alert fatigue. Using Natural Language Processing (NLP) with ever-growing artificial intelligence (AI) capabilities, surveillance solutions may be able to extract information from daily documentation and refine the algorithms for “cardiogenic shock alert” systems.

### Role for risk prediction in CS

Although multiple risk prediction scores have been published for AMI-CS, none are widely used in contemporary clinical practice (172). While the IABP-SHOCK II score is applicable to only AMI-CS patients, the Inova Heart and Vascular Institute (IHVI) score, CardShock risk score, and SCAI staging systems may be used more broadly in other forms of CS (Table 4) (2, 11, 19, 37, 173). Despite availability of multiple risk prediction tools, development of a unified scoring system that is capable of early prognostication to inform not only the therapeutic decisions, but also encompassing risk prediction at multiple time points and in different forms of CS has been challenging. Ideal risk scores for assessing patients with CS should be contemporary, specific to etiologies and phenotypes, and consider the evolving nature of the disease. These scores should address subphenotypes, such as acute *de novo* HF-CS as compared to acute-on-chronic HF cases, which may present differently. An “ideal risk score” should use readily available metrics at the initial presentation and incorporate serial data collected during the index hospitalization to refine prognostic estimates. It should also account for the patient’s response to initial treatment and potentially include novel and clinically actionable variables. For this reason, RCT populations for score derivation may not be ideal. Large, real-world multicenter CS registries may be a better source for generating risk scores. These scores should be easy to calculate at the bedside and incorporate clinical, biochemical, and hemodynamic parameters of interest. Additionally, the concept of a “futility score” is proposed to rapidly identify patients unlikely to survive, facilitating appropriate resource allocation and early engagement of palliative and hospice care (172).

**Table 4:** Cardiogenic shock risk prediction tools.

Risk scores	Year	Variables	
CardShock (19)	2015	• Age >75 • ACS etiology • Prior MI or CABG	• Confusion at presentation • LVEF <40% • Lactate level
IABPSHOCK II (173)	2017	• Age >73 • Prior stroke • Glucose >191 mg/dl	• Creatinine >1.5 mg/dl • Lactate >5 mmol/L • TIMI flow <3
IHVI (11)	2019	• Age ≥71 • Diabetes • Dialysis • Vasopressor use (≥ 36 h)	• Lactate ≥3 mg/dl • CPO <0.6 W • PAPi <1.0
CLIP (180)	2021	• Cystatin C • Lactate	• Interleukin-6 • N-terminal-pro-B-type natriuretic peptide
SCAI (37)	2022	Multiple clinical and lab parameters that risk stratify into 5 stages A to E, with cardiac arrest and arrhythmia modifier	

ACS, acute coronary syndrome; CABG, coronary artery bypass grafting; CPO, cardiac power output; LVEF, left ventricular ejection fraction; MI, myocardial infarction; PAPi, pulmonary artery pulsatility index; TIMI, thrombolysis in myocardial infarction score.

### Utility of machine learning and artificial intelligence approaches to CS

Machine learning approaches are rapidly emerging to elucidate the mechanisms of CS. A retrospective analysis of 1,959 patients with CS from two separate CS registries (Cardiogenic Shock Working Group and Danish Retroshock MI Registry) used machine learning approaches to identify and consecutively validate three distinct phenotypes or clusters of CS patients: “non-congested (I),” “cardiorenal (II),” and “Cardiometabolic (III).” (89) Phenotype I patients, the non-congested profile, were more likely to have lower heart rates, higher blood pressures, and lower filling pressures (right atrial pressure and pulmonary capillary wedge pressure) relative to the other phenotypes. Phenotype II patients, the cardiorenal profile, were older and more likely to have comorbidities of diabetes mellitus, chronic renal insufficiency, and hypertension. These patients were at higher odds of displaying elevated mean pulmonary artery pressures and left-sided filling pressures and consequently decreased glomerular filtration rates (GFR). This group had two- to three-fold mortality compared to phenotype I. Lastly phenotype III patients, the cardio-metabolic profile, were the sickest of all the phenotypes. This group exhibited significant elevations in biomarkers such as lactic acid and aminotransferases. Clinically, they had more profound hypotension, tachycardia, elevations in right atrial pressures, decrease in cardiac power output and cardiac index. This group had up to five-fold increase in mortality relative to phenotype I (89). Although the clustering algorithm used baseline laboratory values, the identified phenotypes showed consistent clinical differences in demographics, comorbidities, and hemodynamics. Although these findings have been validated in a CICU population, the clinical utility remains to be determined. (174), It is also unclear how these phenotypes affect prognosis and treatment decisions in contemporary clinical practice. Taken together, these findings illustrate that individualized risk stratification based on etiology and unique CS phenotype may be useful in guiding therapy and improving clinical outcomes.

### Designing randomized clinical trials in cardiogenic shock

Although RCTs remain the gold standard in CS, there have been numerous challenges to randomization of care and trial enrollment in critically ill CS patients. Potential reasons for these negative results in RCTs, especially in AMI-CS, include but are not limited to: (1) selection bias and failure to account for population heterogeneity; (2) inclusion of patients who are unlikely to benefit, including many cardiac arrest patients; (3) slow enrollment and low statistical power; (4) inadequate matching to shock severity and/or severity of illness; (5) enrollment after onset of multiorgan failure and irreversible sequelae of hemometabolic shock (175, 176). Observational studies have yielded conflicting results, albeit with limitations due to residual confounding, and sometimes contrary to the results of RCTs. Conducting RCTs in patients with AMI-CS is challenging due to the need for rapid informed consent from critically ill patients or their legal representatives as well as physician biases regarding equipoise with respect to the use of MCS devices. In the United States, there is a statute for Exception from Informed Consent (EFIC) under Title 21, Code of Federal Regulations, Section 50.24 (21 CFR 50.24) which allows investigators to perform “investigations involving human subjects who have a life-threatening medical condition that necessitates urgent intervention (for which available treatments are unproven or unsatisfactory), and who, because of their condition (e.g., traumatic brain injury) cannot provide informed consent.” This has not been commonly employed in CS trials to date but will be critically examined in upcoming CS trials including Recover IV.

Notably, negative findings from RCTs may mask positive treatment effects in specific subgroups, emphasizing the importance of evaluating important interactions and heterogeneity of treatment effect in subgroups in larger RCTs (175). Large-scale multinational RCTs with sufficient statistical power and inclusion of a population that closely represents real-world CS cases are crucial. While the promise of registry-based RCTs remains to be realized, there are several opportunities to heeds the lessons of recent RCTs in designing pragmatic and adaptive studies to move the field forward (176). There is a need for robust research infrastructure, inclusion of diverse patient populations, and engagement of key stakeholders in creating prospective studies with cluster randomized or stepped-wedge trial designs.

### Survivorship in cardiogenic shock

Survivors of an acute episode of CS are susceptible to the long-term ramifications of this critical illness, as compromised functional status and diminished quality of life remain pervasive (177). However, evidence suggests that despite these challenges, significant proportions of survivors maintain relatively favorable functional status and quality of life, underscoring the potential for meaningful recovery. In the SHOCK trial, 87% of patients who survived for one-year had NYHA functional class I or II (178). Another CS study estimated the total life years gained was 410 years for the 230 patients with approximately $10,000 per life year gained (24). In another series of CS patients treated with ECMO, nearly all patients managed to perform daily activities and even return to gainful employment at 1-year post-index hospitalization, attesting to the potential for successful rehabilitation (179). Recently, the IHVI Shock Registry reported a 57% survival at 1 year for HF-CS and 47% for AMI-CS based on the three year study period from 2017 to 2019 (17). Higher SCAI stage D/E and age were independently associated with 1-year mortality for both AMI-CS and HF-CS with acute decompensated heart failure as the most common cause of readmission within 30 days of discharge, irrespective of etiology. Future trials such as HALO-Shock [NCT04419480] will leverage remote hemodynamic monitoring such as Abbott’s proprietary CardioMEMS technology, an implantable pulmonary artery pressure monitoring platform, to intervene early after discharge for patients with HF-CS to assess its impact on mortality, rehospitalizations, quality of life, and biomarkers (113). Understanding and improving long term outcomes after critical illness are vitally important and efforts to promote convalescence and full recovery after critical illness, especially during the vulnerable transition phase from ICU to post discharge follow-up, should receive greater consideration (177).

## Conclusion

CS is a complex, multifactorial, hemo-metabolic syndrome with increasing prevalence in the modern CICU. Despite significant advances in medical and device-based therapies, CS remains associated with high morbidity and mortality, especially with increasing incidence of HF-CS. Due to the protean clinical presentations and treatment options, managing these critically ill patients requires a comprehensive, standardized, multidisciplinary team-based approach, using a regionalized system of care. Future efforts should focus on phenotyping of CS and enhancing long-term outcomes in CS survivors, leveraging registry based RCTs and multicenter shock registries to determine the optimal timing for initiating therapeutic strategies, weaning tMCS, and bridging to destination strategies including durable LVAD, heart transplant, and native myocardial recovery.

## Supplementary material

Supplemental Figure 1 Schematic representation of multidisciplinary cardiogenic shock (CS) team activation through a 1-call “shock line” to gather physicians for multidisciplinary consultation and decision making. This “shock team” can be activated by any department not only within the hospital but also across the region, to provide appropriate management for CS. CI, cardiac index; CO, cardiac output; CPO, cardiac power output; DNR, Do Not Resuscitate order; dPAP, diastolic pulmonary arterial pressure; MAP, mean arterial pressure; PAPi, pulmonary arterial pulsatility index; PCWP, pulmonary capillary wedge pressure; pMCS, percutaneous mechanical circulatory support; sPAP, systolic pulmonary arterial pressure; other abbreviations as in Figures 1–4.

Supplemental Figure 2 Schematic representation of the care pathways in the upstream and critical care management of patients with acute myocardial infarction (AMI) and heart failure (HF) cardiogenic shock at Inova Schar Heart and Vascular Institute. CI, cardiac index; CO, cardiac output; CPO, cardiac power output; DNR, Do Not Resuscitate order; dPAP, diastolic pulmonary arterial pressure; MAP, mean arterial pressure; PAPi, pulmonary arterial pulsatility index; PCWP, pulmonary capillary wedge pressure; pMCS, percutaneous mechanical circulatory support; sPAP, systolic pulmonary arterial pressure; other abbreviations as in Figures 1–4.
