# Supplementary figures and images for: Contemporary approach to cardiogenic shock care: a state-of-the-art review
Source: Front Cardiovasc Med. 2024 Mar 13;11:1354158. doi: 10.3389/fcvm.2024.1354158 (PMC10965643; doi:10.3389/fcvm.2024.1354158)

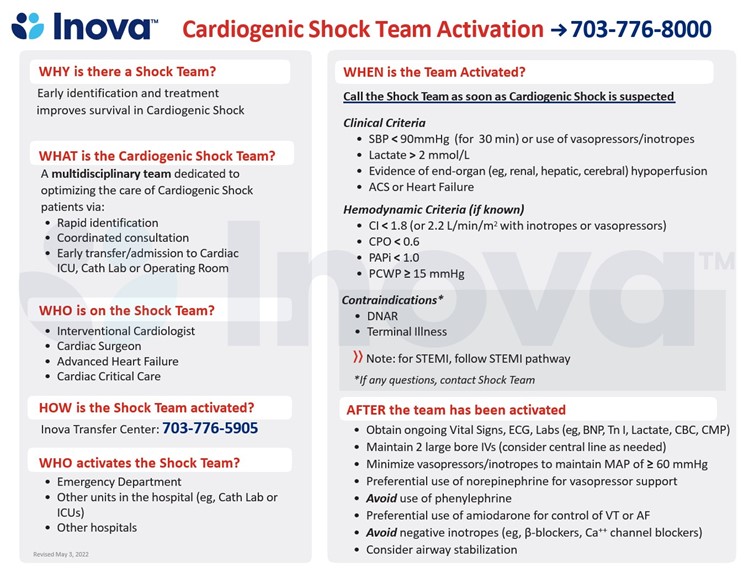

Supplement: Supplemental Figure 1 — Schematic representation of multidisciplinary cardiogenic shock (CS) team activation through a 1-call “shock line” to gather physicians for multidisciplinary consultation and decision making. This “shock team” can be activated by any department not only within the hospital but also across the region, to provide appropriate management for CS. CI, cardiac index; CO, cardiac output; CPO, cardiac power output; DNR, Do Not Resuscitate order; dPAP, diastolic pulmonary arterial pressure; MAP, mean arterial pressure; PAPi, pulmonary arterial pulsatility index; PCWP, pulmonary capillary wedge pressure; pMCS, percutaneous mechanical circulatory support; sPAP, systolic pulmonary arterial pressure; other abbreviations as in Figures 1–4. [file Image1.jpeg]

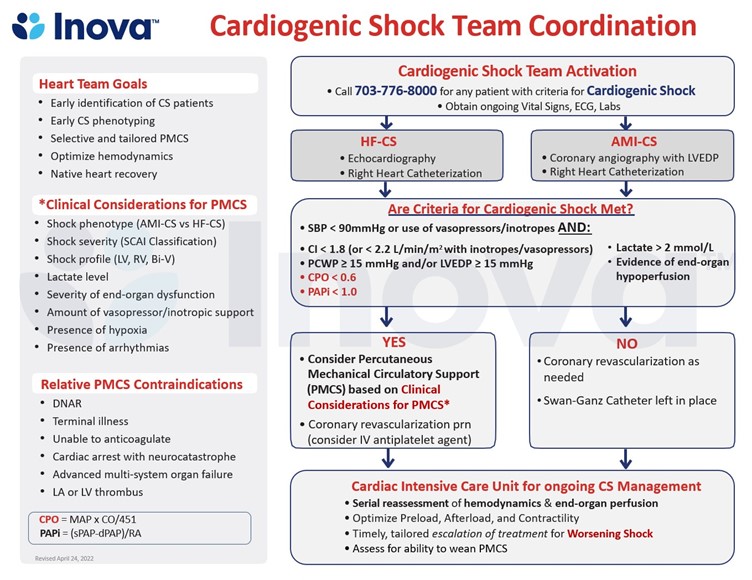

Supplement: Supplemental Figure 2 — Schematic representation of the care pathways in the upstream and critical care management of patients with acute myocardial infarction (AMI) and heart failure (HF) cardiogenic shock at Inova Schar Heart and Vascular Institute. CI, cardiac index; CO, cardiac output; CPO, cardiac power output; DNR, Do Not Resuscitate order; dPAP, diastolic pulmonary arterial pressure; MAP, mean arterial pressure; PAPi, pulmonary arterial pulsatility index; PCWP, pulmonary capillary wedge pressure; pMCS, percutaneous mechanical circulatory support; sPAP, systolic pulmonary arterial pressure; other abbreviations as in Figures 1–4. [file Image2.jpeg]
